# A Serious Game for Massive Training and Assessment of French Soldiers Involved in Forward Combat Casualty Care (3D-SC1): Development and Deployment

**DOI:** 10.2196/games.5340

**Published:** 2016-05-18

**Authors:** Pierre Pasquier, Stéphane Mérat, Brice Malgras, Ludovic Petit, Xavier Queran, Christian Bay, Mathieu Boutonnet, Patrick Jault, Sylvain Ausset, Yves Auroy, Jean Paul Perez, Antoine Tesnière, François Pons, Alexandre Mignon

**Affiliations:** ^1^ Percy Military Teaching Hospital Department of anesthesiology and intensive care French Military Health Service Clamart France; ^2^ Marie Lannelongue Surgical Center Department of anesthesiology and intensive care Le Plessis Robinson France; ^3^ Bégin Military Teaching Hospital Department of visceral and vascular surgery French Military Health Service Paris France; ^4^ Military Medical Center 8ème Régiment Parachutiste d’Infanterie de Marine French Military Health Service Castres France; ^5^ Military Medical Center 3ème Régiment Parachutiste d’Infanterie de Marine French Military Health Service Carcassonne France; ^6^ French Military Health Service Academy Tactical Care Training Department French Military Health Service Paris France; ^7^ École du Val-de-Grâce French Military Health Service Academy French Military Health Service Paris France; ^8^ Hospital and Research Division Head Office of French Military Health Service French Military Health Service Vincennes France; ^9^ iLUMENS Department of Simulation Université Sorbonne Paris Cité Paris France

**Keywords:** serious games, forward combat casualty care, care under fire, interdisciplinary collaboration, simulation, medical simulation, virtual simulation, training, education, assessment

## Abstract

**Background:**

The French Military Health Service has standardized its military prehospital care policy in a ‘‘Sauvetage au Combat’’ (SC) program (Forward Combat Casualty Care). A major part of the SC training program relies on simulations, which are challenging and costly when dealing with more than 80,000 soldiers. In 2014, the French Military Health Service decided to develop and deploy 3D-SC1, a serious game (SG) intended to train and assess soldiers managing the early steps of SC.

**Objectives:**

The purpose of this paper is to describe the creation and production of 3D-SC1 and to present its deployment.

**Methods:**

A group of 10 experts and the Paris Descartes University Medical Simulation Department spin-off, Medusims, coproduced 3D-SC1. Medusims are virtual medical experiences using 3D real-time videogame technology (creation of an environment and avatars in different scenarios) designed for educational purposes (training and assessment) to simulate medical situations. These virtual situations have been created based on real cases and tested on mannequins by experts. Trainees are asked to manage specific situations according to best practices recommended by SC, and receive a score and a personalized feedback regarding their performance.

**Results:**

The scenario simulated in the SG is an attack on a patrol of 3 soldiers with an improvised explosive device explosion as a result of which one soldier dies, one soldier is slightly stunned, and the third soldier experiences a leg amputation and other injuries. This scenario was first tested with mannequins in military simulation centers, before being transformed into a virtual 3D real-time scenario using a multi-support, multi–operating system platform, Unity. Processes of gamification and scoring were applied, with 2 levels of difficulty. A personalized debriefing was integrated at the end of the simulations. The design and production of the SG took 9 months. The deployment, performed in 3 months, has reached 84 of 96 (88%) French Army units, with a total of 818 hours of connection in the first 3 months.

**Conclusions:**

The development of 3D-SC1 involved a collaborative platform with interdisciplinary actors from the French Health Service, a university, and videogame industry. Training each French soldier with simulation exercises and mannequins is challenging and costly. Implementation of SGs into the training program could offer a unique opportunity at a lower cost to improve training and subsequently the real-time performance of soldiers when managing combat casualties; ideally, these should be combined with physical simulations.

## Introduction

The survivability of battlefield casualties has recently risen to an unequalled historical level of 90%, compared to 84% in Vietnam and 80% in World War II [1]. Factors most likely related to this improved survivability include technical improvements in body armor and deployment of a comprehensive trauma management system (involving first aid and basic life support on the field and subsequent prompt surgery and critical care). Tactical Combat Casualty Care (TCCC) is now considered as a reference for management of combat casualties at the point of injury. It combines a set of trauma management guidelines designed for use on the battlefield [2,3]. Improved training of soldiers and military caregivers based on the concepts of TCCC plays an important role in the improved survival of combat casualties [4].

In 2007, the French Military Health Service standardized this TCCC concept in its military prehospital care training policy through a specific program entitled ‘‘Sauvetage au Combat’’ (SC, ‘‘forward combat casualty care’’). After delivery of first aid to soldiers in the under fire stage, forward medicalization on the battlefield is one characteristic of the SC. The medical team is sent as close as possible to the casualty at the time of injury. In the SC training program, emphasis is placed on simulations, which are considered a gold standard in team training for improvement of both technical and nontechnical skills, in both civilian and military trauma settings [5-8]. However, the logistics involved in training and testing each French soldier with simulation exercises and mannequins make them challenging and costly. Moreover, there might be important delays between the training period and the actual operations. However, regardless of these delays, knowledge of both adequate procedures and skills has to be maintained.

Computer-based technologies, such as e-learning, massive online open courses, or serious games (SGs), have become increasingly prevalent in education, training, and simulation. SGs are digital simulations similar to video games that are engaging, rewarding, and fun as they simultaneously educate and train [9-10]. SGs use the gamification concept for training applications. Gamification is the application of game-based elements to nongame mechanisms, including education. SGs have drawn much attention over the last decade because they have become more realistic and engaging, owing to technological improvements such as better graphics and new gaming interfaces and gameplays. SGs are also more affordable, owing to their reduced costs of production. Thus, SGs could address a larger population of trainees to increase the frequency of cognitive training, allowing training to occur anytime and anywhere, and can be used to assess retention of procedural skills in a more practical manner and at a lesser cost [11].

Therefore, the French Military Health Service considered in 2014 the development of 3D-SC1, an innovative SG devoted to the training of soldiers for casualty care under fire; 3D-SC1 constitutes the first part of a virtual simulation platform. Combined with the next 3D-SC versions, it will allow training of combat lifesavers (SC2) and nurses and physicians (SC3) for forward combat casualty care applications on the battlefield.

The purpose of this paper is to describe the design and production processes of 3D-SC1 and to discuss its deployment.

## Methods

**Medusims**

Medusims are virtual digital medical simulations using videogame technology to simulate realistic medical situations for training and assessment purposes. They were produced by a French startup, Medusims, created in 2011 [12]. Medusims has already produced 6 SGs in the cardiology area (Staying Alive after cardiac arrest for the general public [www.stayingalive.fr], acute coronary syndrome, atrial fibrillation, and pulmonary embolism), and 2 SGs in the perinatology area (Born to be Alive for the general public [www.borntobealive.fr] and postpartum hemorrhage [12]).

Medusims were used to assess how effective practices are, emphasizing on the importance of having reference material and procedures reflecting real-life scenarios. The trainees used Medusims as an active learning method to familiarize themselves with procedures, without any risk to the patients or casualties. Medusims produced virtual simulations in a 3D studio, using the Unity engine, a multi–operating system and a multiplatform tool allowing trainees to access the digital experiences on personal computers or tablets. Medusims has developed an internal process of in-house production, integrating medicine and technology, to deliver high-quality products, with a production studio panel of game design, human engineering, pedagogic engineering, graphics computing, and 3D animation, working closely with medical and military experts on the topic.

**Preparation of 3D-SC1 project**

The French Military Health Service designed an advisory board of SC experts, most of them being existing members of the Comité Opérationel d’Enseignement du Sauvetage au Combat (COESC, the Operational Committee for SC teaching). Several steps before the beginning of 3D-SC production were also validated, which were as follows.

- Definition of the trainees involved as first player shooter in the experience (soldiers for SC1, health-qualified soldiers, or combat lifesavers for SC2, physicians and nurses for SC3, to come later). In the first version of the experience, there was only 1 person playing (mono user).
- Definition of training objectives for SC1 (survival positioning, compression bandage, tactical tourniquet, and morphine auto-injector use), SC2 (bleeding shock management including intraosseous access, tactical tourniquet assessment, airway, and respiratory management including pneumothorax decompression and cricothyroidotomy), and SC3 (medical trauma management in a hostile remote environment, with tracheal intubation, sedation, early use of tranexamic acid, and vasopressor agents in case of hemorrhagic shock, digital thoracotomy with chest tube insertion, handheld focused assessment with sonography for trauma, and strategies for crisis resource management) [13-15].
- Design of a scenario involving an improvised explosive device (IED) attack on a patrol of 3 soldiers, followed by a second patrol among which was the trainee, allowing the trainee to test the correct procedural skills execution, in the right order and as quickly as possible.
- Test of the realistic features of this scenario in real simulation with mannequins, in order to observe right and wrong actions delivered.
- Design of 2 levels of difficulty—beginner and advanced.
- Design of a correcting grid for scoring and personalized debriefing.
- Design of a rewarding process (bronze, silver, and gold medal graduation) according to the scoring system, integrating time, and actions delivered.
- Design of a gameplay, interface, and the look and feel of the SG.
- Finally, definition of the different animations to be produced, including using 3D motion capture.

Different iterations were developed between the experts and the studio, which allowed production of a beta version in 8 months. After debugging, a final gold version was delivered after 9 months, and it was successfully submitted to the French Army in January 2015.

## Results

### Creation of 3D-SC1

A 10-member expert advisory group was formed to identify training priorities for which 3D-SC1 could be appropriately used. All these experts held local or national roles in organization and training of SC programs. Experienced physicians and soldiers in operational units, with significant experience of deployment in combat zones, also participated. A scenario was created to illustrate a real-life–based experience, the explosion of an IED during a reconnaissance mission. The same panel of experts promoted the scientific validation of the scenario according to the SC French guidelines.

**Figure 1:**
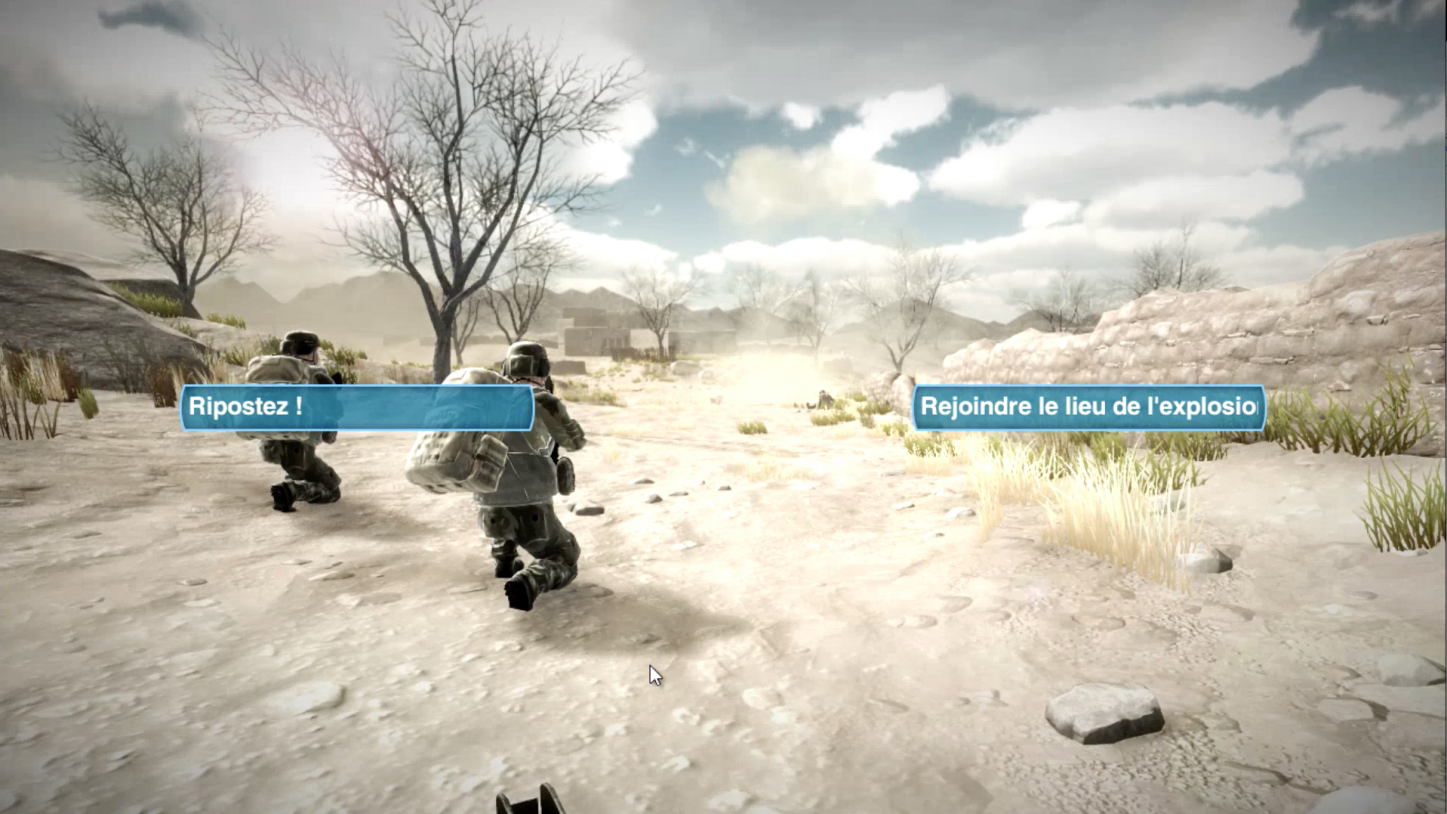
Tactical options in 3D-SC1.

**Figure 2:**
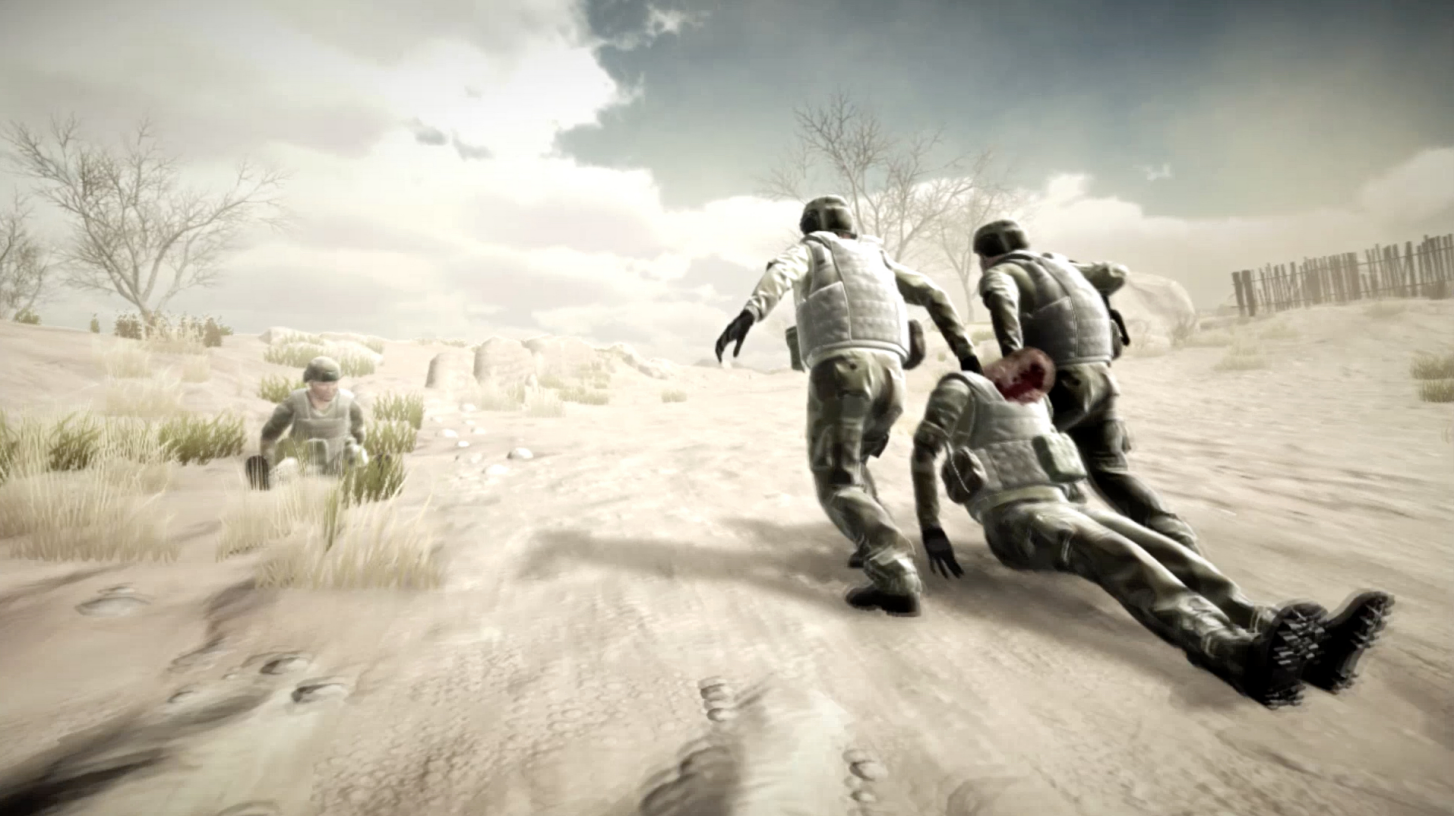
Pick and run in 3D-SC1.

**Figure 3:**
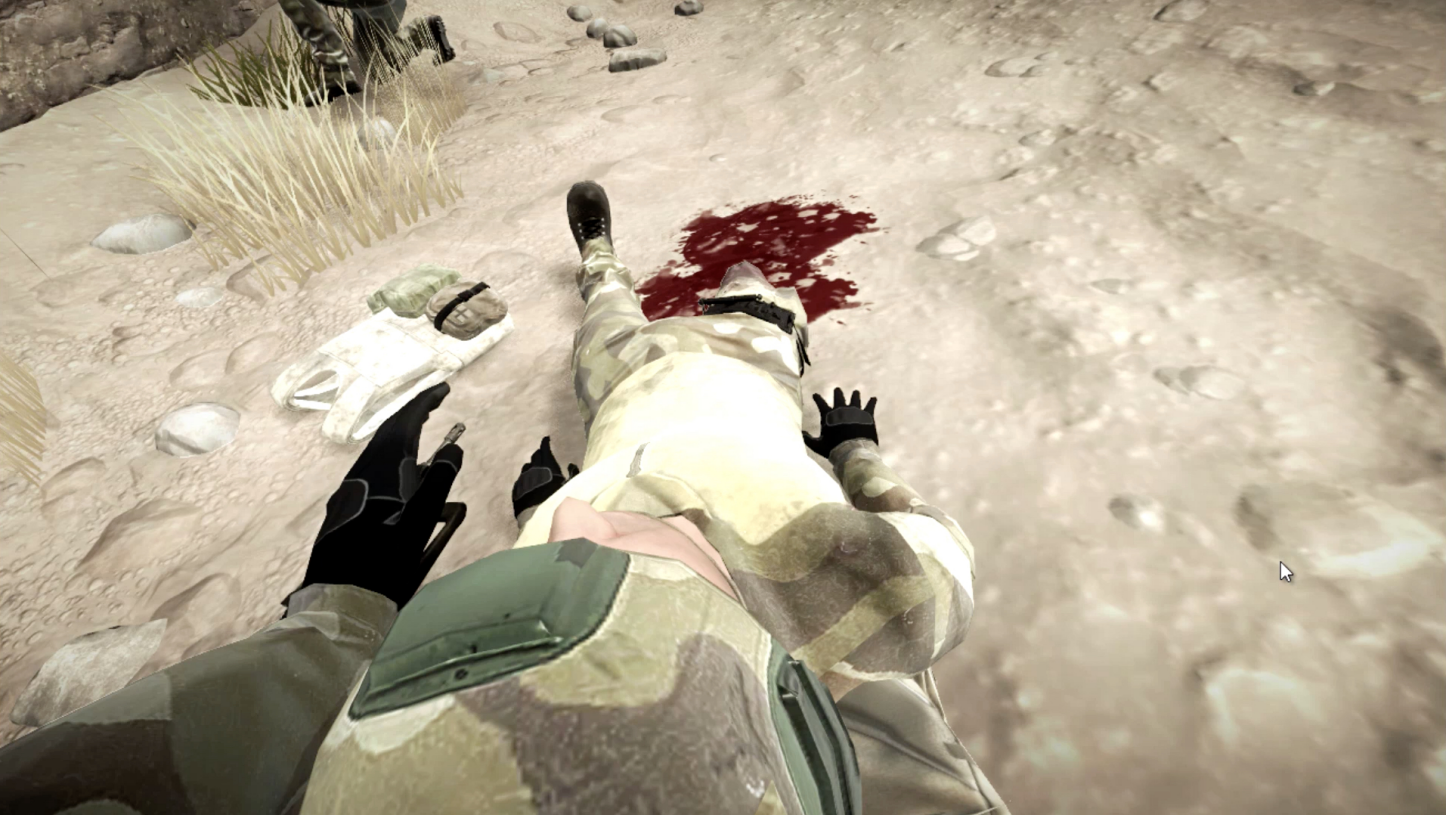
Combat casualty management with tactical tourniquet application and survival positioning in 3D-SC1.

In the 3D-SC1 scenario, the explosion of an IED creates 3 casualties: one is dying, one is slightly stunned; and the last one presents with a traumatic amputation of the limb, difficult breathing, and another hemorrhagic injury under the arm. At the beginning of the experience, the care under fire stage is illustrated by hostile fire after the IED explosion, in a stressful, hostile, and noisy ambience (explosion, firearms shooting, shouts of pain), and an austere setting (desert; Figure 1). The trainee has to choose between several tactical options: return fire, determine which casualty is dead or alive, determine which casualty can return fire, or pick up the casualties and run to cover (Figure 2). When no longer under direct enemy fire, the trainee has to deal with different forward combat life-saving procedures from SC programs (Figure 3): tourniquet application, casualty survival positioning, hemostatic dressing, and morphine auto-injector use. In addition, the trainee has to deal with weapons security, management of personal protective equipment, call for a 9-line medical evacuation (MEDEVAC) and a MIST request (which includes mechanism of injury, type of injury, signs, and treatment given).

### Development and Production of 3D-SC1

**Figure 4:**
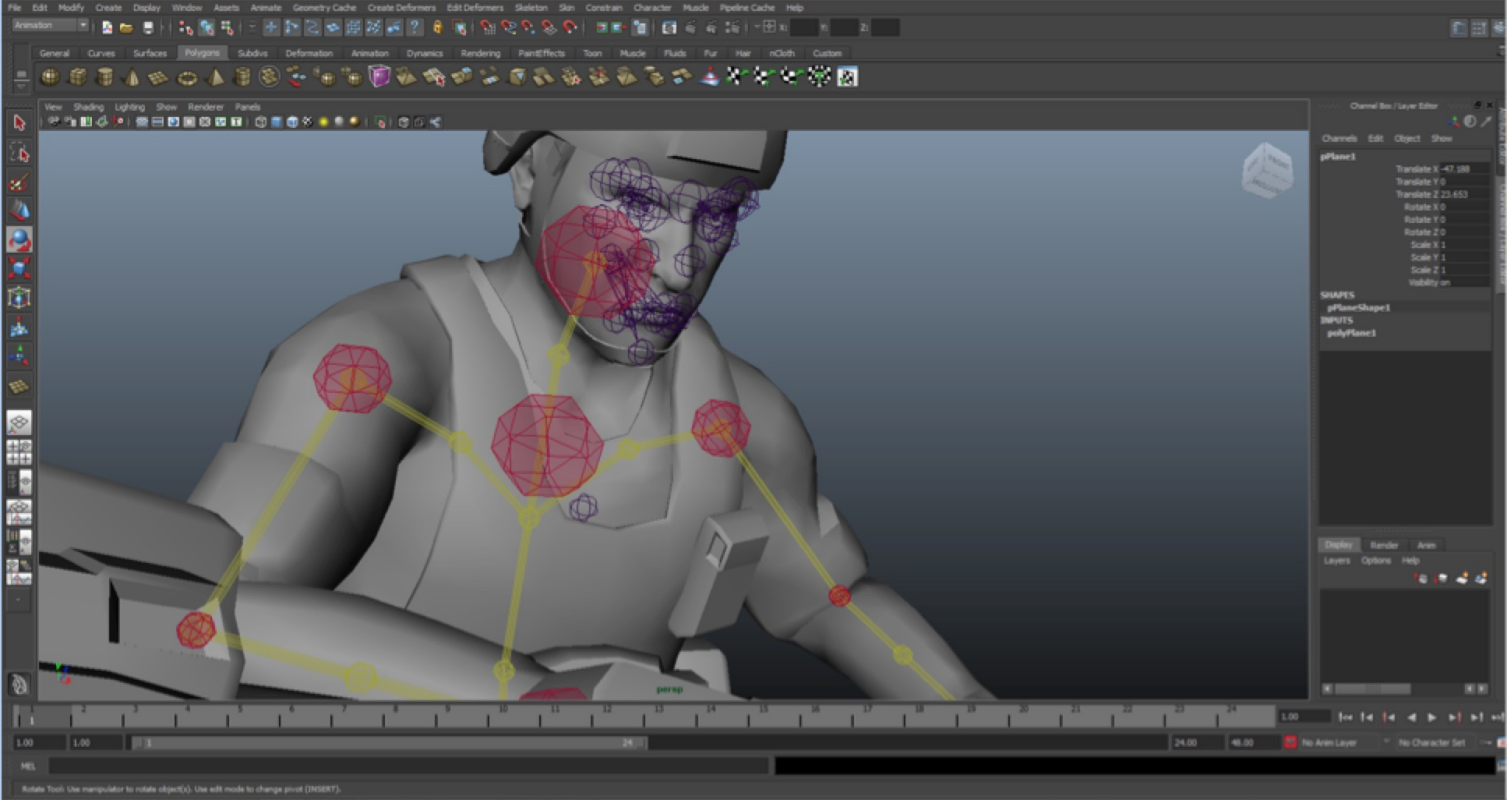
Three-dimensional studio-development of 3D-SC1.

The development of 3D-SC1 was performed in a 3D studio (Figure 4). Particular attention was paid to essential movements and postures, such as tactical tourniquet application, recorded in a motion capture mode to provide to the trainee a high-quality reproduction of the movement in the 3D-SC1 experience. Development included a gamification process where players are challenged to keep on playing to reach the game’s objective. Two levels were defined: beginner and advanced modes.

In case of failure in applying the right procedure at the right time and in the right order, an automatic virtual instructor takes control of the experience and simulates the right procedure. In the advanced difficulty level, the trainee has to face a more restrictive time limit, combined with more challenging procedures: an inefficient first tactical tourniquet and delayed MEDEVAC arrival or frequent changes in tactical context.

**Figure 5:**
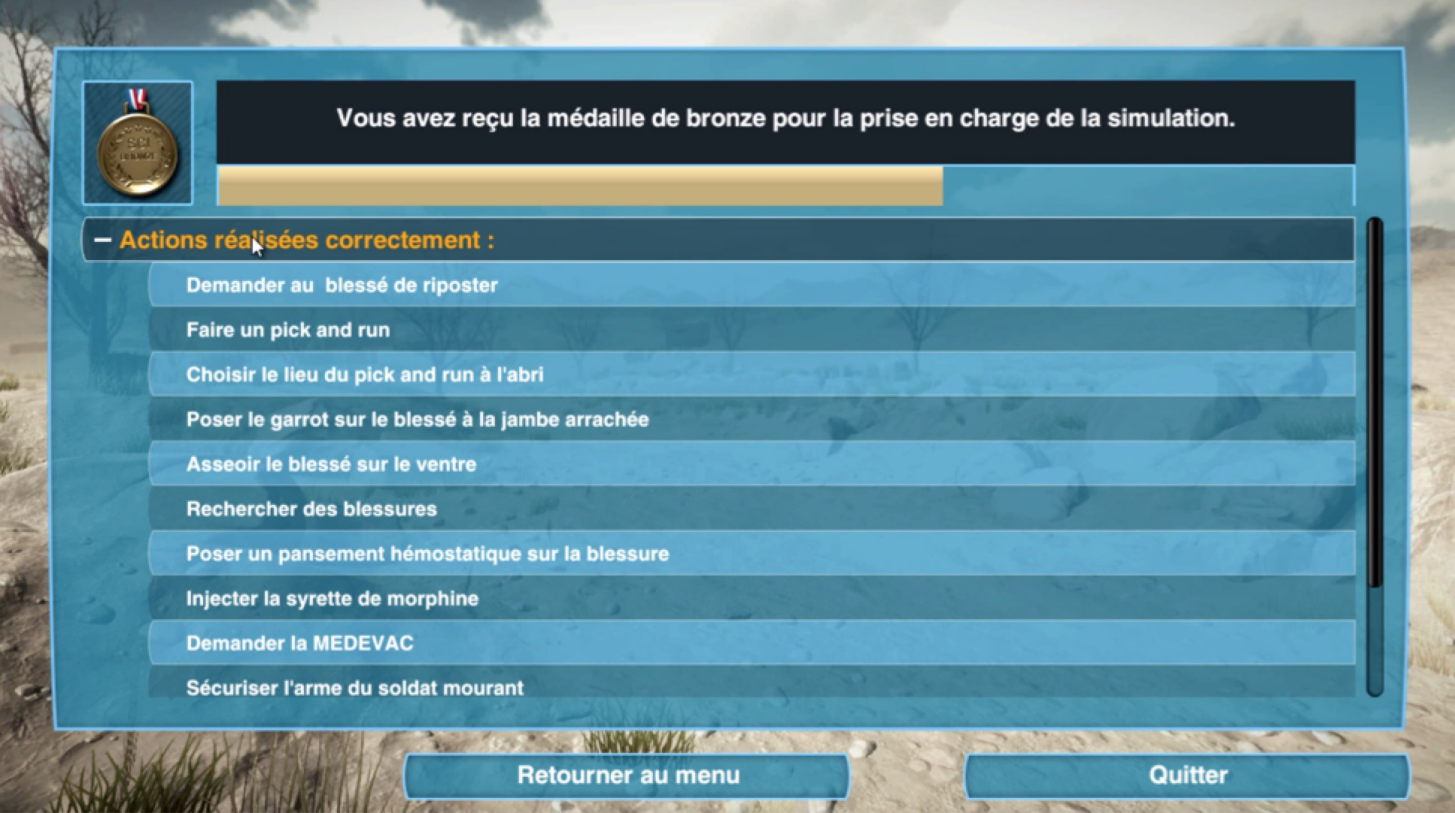
Personalized debriefing at the end of 3D-SC1.

Most importantly, at the end of the 3D-SC1 simulation, a personalized debriefing is proposed (Figure 5), highlighting good performance achieved in the experience, the procedures for which the trainee has to improve, and the missed procedures for which the automatic virtual instructor had to take the control of the experience to perform the procedure [16-17].

Finally, on the basis of a scoring process, the trainee graduates with either a gold, silver, or bronze medal. In case of fatal outcome in the virtual experience due to an inappropriate response of the trainee, an automatic virtual instructor saves the combat casualties, and the trainee obtains nothing but a training certificate. They are invited to participate in the 3D-SC1 experience once again to improve their knowledge in the application of SC procedures.

### Deployment of 3D-SC1

Because SC programs are now completely incorporated into the predeployment training course of all combatants, the diffusion of 3D-SC1 is planned to reach at least all combatants before their deployment in a theater of operations. Successful deployment of 3D-SC1 was conducted in only 3 months and has already reached 84 of 96 (88%) French Army regiments. Solutions for massive implementation of 3D-SC1 include, for now, personal computers and laptops in a basic classroom, with a total of 818 hours of connection in the first 3 months (in hours, for each training center, mean 19.9, [standard deviation 35.2]). However, 3D-SC1 is also scheduled to be deployed on tablets or smartphones. Such an individual deployment of the SG could let the trainees have their own 3D-SC1 experiences anywhere and anytime. The personalized debriefings are planned to be collected in an extensive databank, allowing global statistical analysis, in order to improve the SC training programs according to the results obtained in different groups of trainees.

## Discussion

The development of 3D-SC1, a SG for SC, the French Forward Combat Casualty Care program, has been completed.

Here, we have described an innovative tool for learning and assessing care under fire and its collaborative preparation for design and production by interdisciplinary experts and actors. This led to a fast deployment of the SG, and the integration of a comprehensive collection of data for further assessment of the French SC program.

### Limitations

A major limitation of this study is that no objective data regarding the pedagogic value of the SG were assessed. In fact, we have not measured most benefits of simulation, such as that with 3D-SC1, described in this section. However, a randomized observational comparative study is ongoing. How these SGs could be assessed, as new tools for learning and training, is not clear. When applying the concept of 4 levels in evaluating training programs, as described by Kirkpatrick, we believe that studying preventable deaths as an outcome (the fourth level in the Kirkpatrick model, ie, did the change in behavior positively affect the patient) could be really challenging, especially in a combat setting [18]. We could probably only reach level 3 in the Kirkpatrick model by placing the soldier in a physical simulated environment to assess whether learning is transferred from cognition to skills and attitudes. Other points of discussion include that 3D-SC1 concerns a single-center production. However, as highlighted in the description of the process of creation and production, the development of 3D-SC1 involved a collaborative platform with interdisciplinary actors.

Actually, realization of an SG for SC training is based on the concept that an SG has the potential to increase observance and engagement with training programs in military institutions. There is an increasing interest for medical virtual simulation in numerous settings, including trauma, surgery, anesthesia, emergency medicine, women’s health care, and even patients’ education [19-27].

The benefits of complete virtual training include the ability to create unlimited training scenarios and to repeatedly try and fail in a consequence-free environment. In addition, when training is conducted in a virtual environment, each performed step is considered as essential data. The computer reproduces each movement a trainee makes, and thus, can track and record each and every one of these. Using these data, performance can be automatically quantified to a level that was not achievable ever before [28]. Furthermore, through the processes of scoring and gamification applied in 3D-SC1, the trainee is motivated to improve his personal experience. He also shares his scores with his peers in a competitive and engaging challenge [29-31].

Different virtual simulations have been developed in TCCC programs and several military medical settings, with a very strict adherence to TCCC guidelines depending on the nations’ specific policies [8,28,32-38]. Indeed, 3D-SC1 is following the SC guidelines created by the French Military Health Service Academy (École du Val-de-Grâce) in an official 2007 publication, which has been updated every year by the COESC (the Operational Committee for SC teaching) [13].

Besides SGs, live simulation exercises are the accepted “gold standard” for military preparedness before deployment in a combat zone. However, they are costly and time consuming to organize and may be disrupt local services [39,40]. In contrast, 3D-SC1 *,* used on a standalone computer with a suitable connection for data collection, enables multiple trainees to learn in a real-time, immersive environment, regardless of physical location. Finally, SGs are gradually taking an important place beside physical simulation (high-fidelity manikin simulation combined with casualty simulation moulaged actors), although the incomplete application of strong artificial intelligence, which would allow full mixed-initiative dialogue, can limit their applicability. However, growth in the use of SGs is likely to continue because of their ability to scale inexpensively to large numbers of physically dispersed learners, adapt quickly to prior knowledge and other individual characteristics of learners, and be available anytime and anywhere via a global information infrastructure [41].

A common criticism about SGs is that the dynamic colorful world of a computer game will distract the trainee’s attention from the learning process. However, current soldiers grew up with digital media and have developed a much better aptitude to relate a virtual world to reality. However, this criticism must be addressed for 3D-SC1 to become well accepted. It is also necessary to demonstrate its educational value and its clinical effectiveness in real combat casualty situations.

Virtual environments are not a substitute for hands-on training; they can neither simulate the physical elements of tactical response nor provide training in the dexterity of performing procedures on a casualty. To potentiate existing training, 3D-SC1 can be used as an. For example, it can improve knowledge of adequate procedures before participating in complete military exercises or local SC stages, conducted in lifelike conditions, such as MedicHos Médicalisation en milieu Hostile (Medicalization in a Hostile environment) or ExOSAN Exercice Opération Sanitaire (Exercise for Operation Sanitary) exercises [13,23].

Multiplication of 3D-SC1 scenarios and the next production of SC2 and SC3 could lead to the building of a virtual simulation platform for SC training, incorporated as part of a large military medical simulation-training program combining both SGs and physical simulation. Multiplayer large-scale virtual exercises could be included in predeployment training [42,43]. Finally, upcoming technologies such as augmented reality or haptic simulation could add to the definite 3D-SC program development [34,43,44].

### Conclusions

3D-SC1 is a new SG module, dedicated to the cognitive training of soldiers for forward combat casualty care, during the care under fire stage. The development of 3D-SC1 involved a collaborative platform with interdisciplinary actors, including forward combat casualty care experts (the French SC), programmers, and game designers. The creation and production of 3D-SC1 outlines the applicability and acceptability of using virtual environments for training in SC procedures in dedicated scenarios. This type of simulation could easily be adapted to address different training needs of SC1 (such as urban combat or convoy attacks), SC2, and SC3 (such as combat casualty management during the tactical field care phase and preparation for casualty evacuation). 3D-SC1, actually designed for the French Army, could also find many applications to train people involved in the management of trauma casualties in other settings such as tactical emergency care, disaster and crisis management, or mass casualty events. Further possibilities related to the application of 3D-SC1 are unlimited. The observational comparative trial addressing its educational value is ongoing, and its integration in a mandatory official military degree validation is the next step [23,45-51].

## Abbreviations

COESC: Comité Opérationel d’Enseignement du Sauvetage au Combat
MEDEVAC: medical evacuation
SC: Sauvetage au Combat
TCCC: Tactical Combat Casualty Care
